# Clinical experience with woven and parallel hamstring-tendon anterior cruciate ligament reconstruction

**DOI:** 10.1186/s43019-019-0002-6

**Published:** 2019-07-03

**Authors:** Divyanshu Goyal, Sandeep Yadav, Vidyasagar JVS

**Affiliations:** 1 Fellowship trainees in arthroscopy and sports medicine, B-21, Vaishali nagar, Jaipur, Rajasthan India; 2 Head of the Department of Orthopaedics and Arthroscopy and Sports Medicine, Hyderabad, India

**Keywords:** ACL reconstruction, Hamstring tendon graft, Parallel strand, Weave technique, Graft preparation method

## Abstract

**Purpose:**

The purpose of this study was to determine the effect of the weave technique for hamstring graft preparation on the diameter of the prepared graft, functional outcome, and need for harvesting of semitendinosus and gracilis (ST + G) or semitendinosus alone (ST).

**Materials and Methods:**

This retrospective study evaluated 340 patients who underwent arthroscopic anterior cruciate ligament (ACL) reconstruction from January 2013 to December 2015. Our protocol for graft preparation is that the graft length must be a minimum of 8 cm and the diameter must be between 7 and 10 mm. The parallel-graft preparation technique was used in 189 patient and the weave technique was used in 151 patients. Outcome was measured by using stress radiographs and International Knee Documentation Committee (IKDC) 2000 score.

**Results:**

In the parallel-graft preparation group, ST + G was used in 99 patients and ST was used in 90 patients. In the weave-graft preparation group, ST + G was used in 38 patients and ST alone was sufficient in 113 patients. The need for G harvest was less in the weave-technique group (*p* < 0.0001). There was no statistically significant difference at 2 years of follow-up in stress laxiometry, IKDC 2000 scores and rerupture rates between the two groups.

**Conclusion:**

The weave technique helps to reduce the need for G harvest without compromising functional outcome.

Level of evidence IV.

## Introduction

Reconstruction of the anterior cruciate ligament (ACL) is recommended for the prevention of instability, further intra-articular disease, and recurrent injury in the ACL-deficient knee [1]. Because of the reported lower donor-site morbidity, a semitendinosus (ST) or combined ST and gracilis (STGR) tendon graft is commonly used for reconstruction of the ruptured ACL [2]. Evaluation of patient muscle strength after ACL reconstruction is used to determine whether the patient can safely return to their pre-injury activity level [3]. Most of the studies that have evaluated autologous ST and STGR grafts have focused on postoperative graft remodeling and knee-flexor strength [4]. Studies examining the knee-flexion strength of patients after ACL reconstruction have noted very small or no deficits in peak torque after ST or STGR harvest [5, 6], but some authors have reported a persistent deficiency in flexor strength after surgery [7]. There have also been reports evaluating the rotation torque of the knee. A growing body of evidence indicates that there are large deficits in the internal rotation strength, a significant weakness of hamstring muscle strength at high knee-flexion angles, and a significantly lower standing knee-flexion angle after STGR harvest, which has led some authors to recommend the harvest of only the ST tendon whenever possible [8–10].

Biomechanical studies using animal tendons to determine the effect of braiding or twisting on initial graft strength and stiffness have not yielded clear conclusions. A study done by Kim et al. on human tendons show that twisting and braiding reduces the tensile strength and stiffness of human hamstring tendon grafts used for ACL reconstruction [11]. However, no study is available regarding the impact of the weave technique on strength and stiffness of the hamstring tendon graft used for ACL reconstruction with proper pretensioning of the graft.

The purpose of this study was to determine the effect of the weave technique for hamstring graft preparation on the diameter of the prepared graft, the need for harvesting of ST and G or ST alone, and functional outcome. Our hypothesis is that the weave technique and parallel-graft technique do not affect the final graft diameter and the need for harvesting of the G tendon.

## Materials and methods

After obtaining Ethical Committee approval, we retrospectively evaluated 340 patients who underwent arthroscopic ACL reconstruction from January 2013 to December 2015. Out of these, 189 patients were treated using the parallel-graft technique and 151 patients using the weave technique. ACL reconstructions before October 2014 were done using the parallel-graft preparation technique and thereafter the weave-graft preparation method was used. For ACL reconstruction, hamstring graft tendons (ST with G or ST alone) were used. For graft preparation, we followed the protocol of our institution: the graft length must be a minimum of 8 cm and the diameter must be between 7 and 10 mm. In patients who had a graft diameter less than 7 mm after preparation by either method, we harvested the G. We excluded patients who had a partial ACL tear or a meniscal tear (grade 3), underwent double-bundle ACL reconstruction, in whom an allograft was used or any method of fixation used other than aperture fixation, and who had multi-ligamentous injury.

## Surgical technique

Under spinal or epidural or general anesthesia, the knee is examined for ligament injury using Lachman’s test, the anterior drawer test, pivot shift test, valgus and varus stress test in full extension and in 30^°^ of knee flexion, and posterior drawer test. After applying a tourniquet, the operated part was cleaned and draped. A diagnostic arthroscopy was performed using standard anterolateral and anteromedial portals.

Hamstring tendons are identified at an average of 2 cm distal to the joint line and 2 cm medial to the tibial tubercle. A longitudinal incision over the anteromedial tibia is made, and dissection is carried through the subcutaneous tissue till the sartorius fascia is reached. The fascia is palpated to identify the underlying ST and G tendons. The tendons are blend together at their insertion site on the tibia and the interval between them is more distinct proximally and posteriorly. The sartorius fascia is then incised over this interval in the line of tendons. A right-angle clamp is used to isolate the tendons. After the vincular attachments are cut under visual control, the ST and G tendons are harvested with a closed-type tendon stripper.

**Fig. 1:**
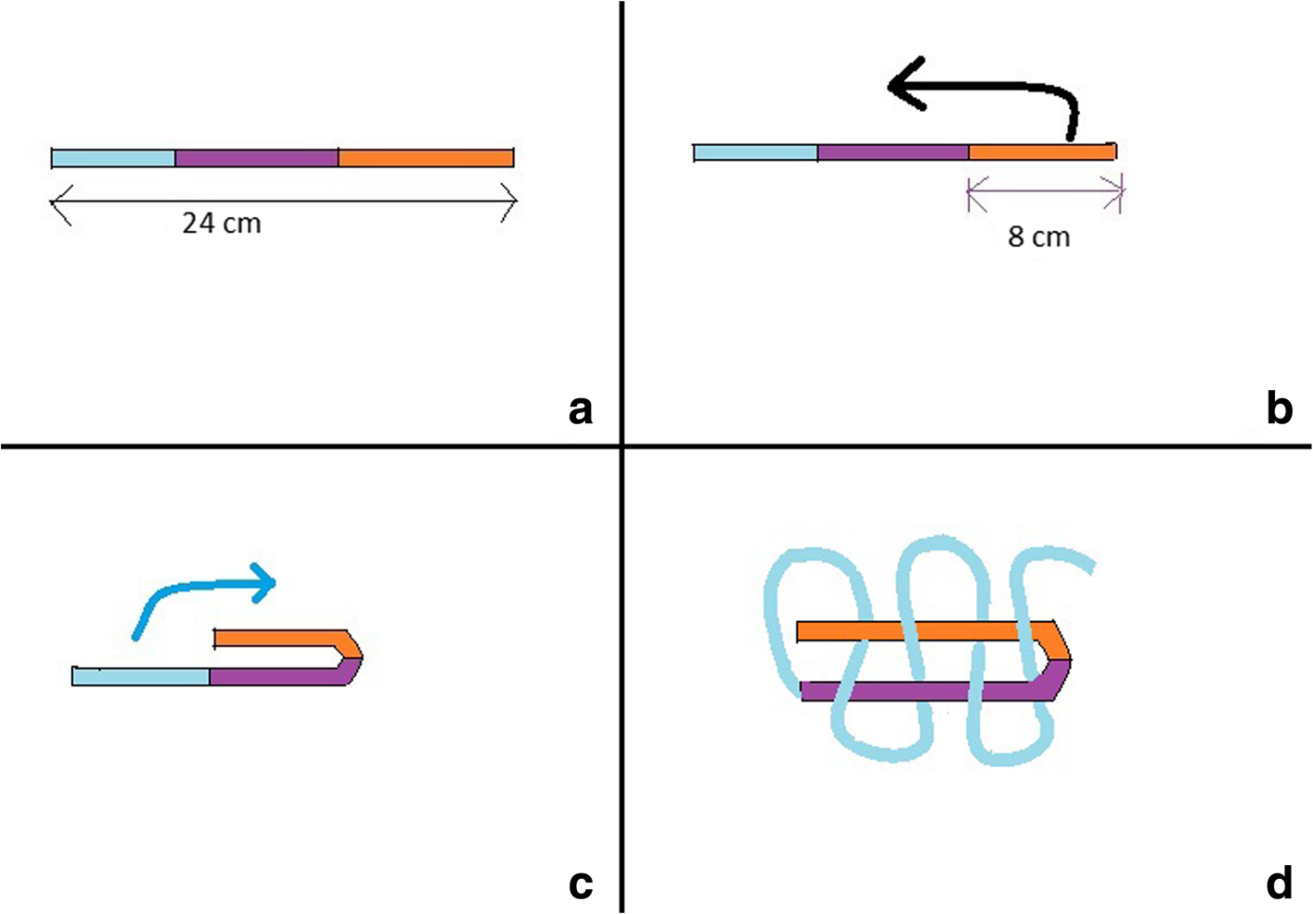
Graft preparation in the weave technique. **a** total length of harvested ST graft. **b** graft is bent at around 8 cm of length. **c** remaing 8 cm is bent in weave pattern. **d** final appearance after weaving

**Fig. 2:**
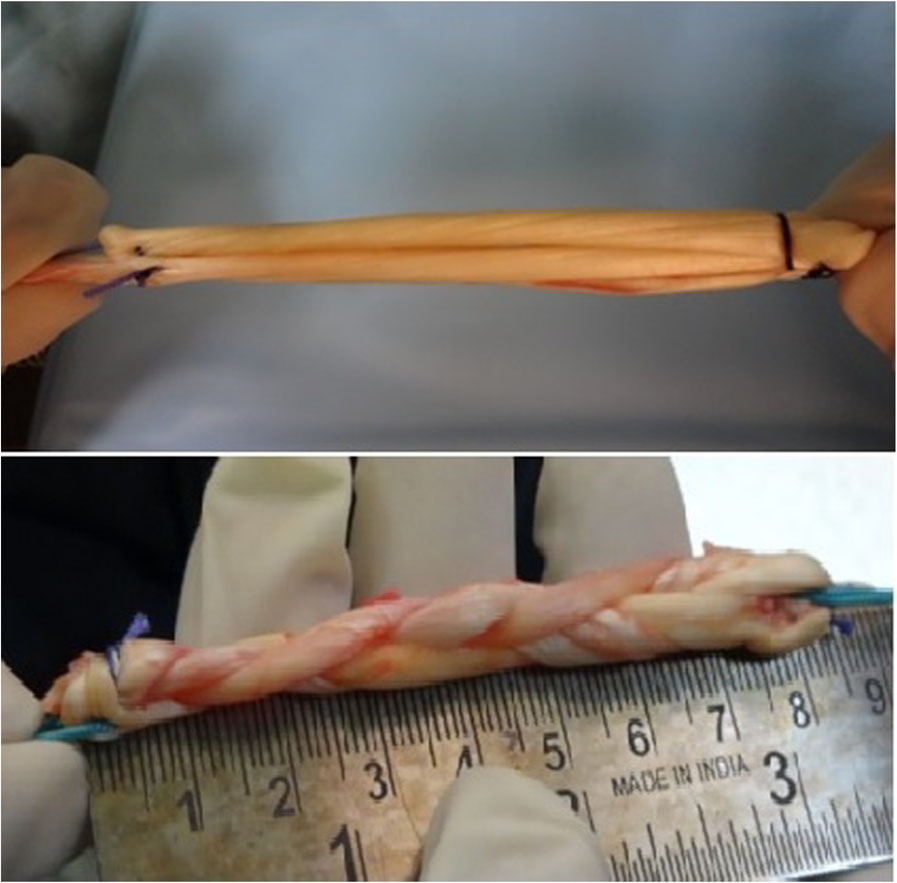
Prepared parallel-strand and woven-hamstring grafts

**Fig. 3:**
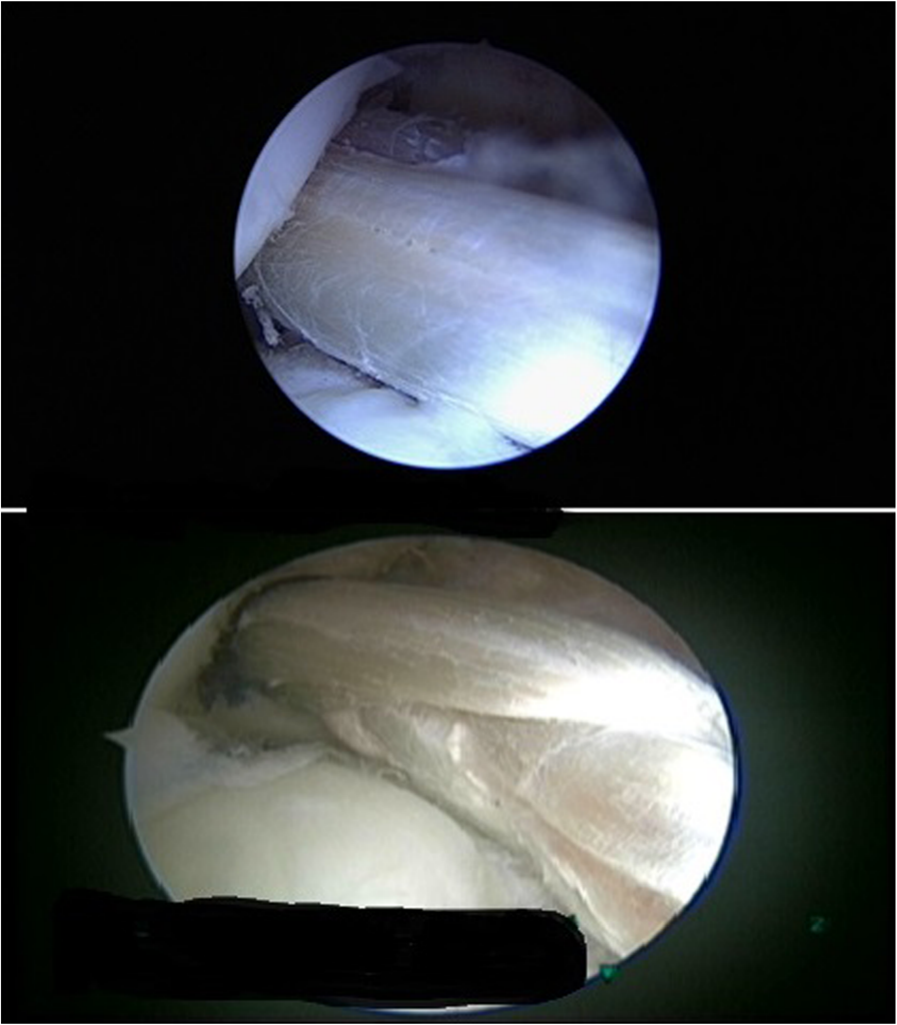
Arthroscopic images of the parallel graft and woven graft inside the knee joint

After harvesting ST + G or ST tendons, attached soft tissue is removed with the non-cutting side of a surgical knife. The length of the harvested graft is measured and is usually about 240 mm for the ST. In the parallel-graft preparation method, one end of the graft (distal of tendon) is sutured with a 1–0 vicryl suture and fixed to a clamp (C1) on the graft preparation platform, the graft is bent at about 80 mm and secured to another clamp (C2) with a 1–0 vicryl suture. Another 80 mm of the graft (proximal of tendon) is bent again at C1 and fixed to C2 after pretensioning and stitched with a 1–0 vicryl suture. Graft diameter should be between 7 and 10 mm. So if it is required to increase the diameter, the G tendon is also harvested and prepared over the earlier graft in a similar manner. This forms the quadrupled or pentavalent hamstring tendon graft. In the weave technique, the initial part of graft preparation is the same. The two thirds of the graft are bent in a similar fashion and clamped to the two respective clamps as stated earlier. These are parallel. Now the remaining third (80 mm) is woven over these two parallel strands and stitched to clamp C2 after pretensioning (Figs. 1, 2, 3). If the graft diameter is less than 7 mm, then the G is harvested.

An accessory anteromedial portal is used for femoral tunnel preparation in more than 100° knee flexion. The graft is fixed with aperture fixation (tunnel mouth fixation/interference screw) using screws (titanium or biodegradable).

Patients were given intravenously administered antibiotics for 1–2 days postoperatively. Ankle and foot movements, static quadriceps exercises, knee range of motion, and straight leg raising were started as soon as the patients recovered from anesthesia. Mobilization walking with walker support was started the next day with priority focussed on the recovery of full extension. Wound inspection was done on the second postoperative day and patients were discharged with orally administered antibiotics and analgesics for 5 days. Stitch removal was performed 10 days postoperatively. All patients underwent the same rehabilitation program till they achieved a full range of motion at the knee joint and 5/5 power for quadriceps and hamstrings.

Follow-up was done at the time of stitch removal and 6 months and 2 years postoperatively. Patients who were lost to follow-up were excluded from the study. Only those patients who completed full rehabilitation were included in the study. Outcome was measured by using the International Knee Documentation Committee (IKDC) 2000 score (subjective), radiographic stress laxiometry (objective), and rerupture rate of the reconstructed ACL. In stress radiography, the lateral view of the knee joint at 90° of flexion was taken by applying the anterior drawer stress simultaneously (as the anterior drawer test is routinely done at 90° of flexion). Anterior tibial translation was then measured in millimeters on these radiographs. Flexor strength was checked clinically preoperatively and periodically postoperatively by asking the patient to perform prone knee bending with a strap weight attached just proximal to the ankle (maximum load and standard set of 30 repetitions).

The data were analysed by software MEDCALC. Group comparisons were done using a *t* test and a *p* value of less than 0.05 was considered significant.

## Results

**Fig. 4:**
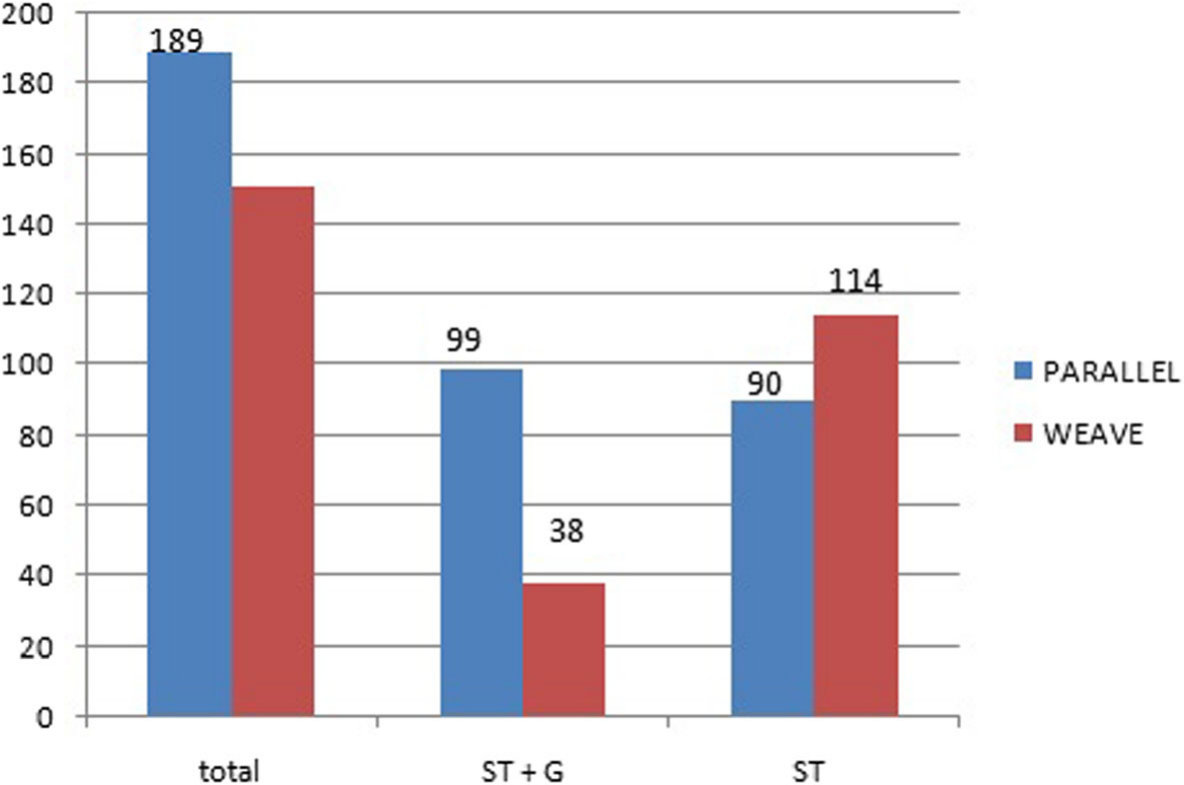
Bar diagram representing the decreased use of the gracilis tendon in the weave-graft preparation technique

In the parallel-graft preparation group, the male and female patient number was 165 and 24, respectively (Fig. 4). Their mean age was 32.2 years (range, 20–45 years) (Table 1). The hamstring tendons in the form of ST + G were used in 99 patients (52.38%) and only ST, in 90 (47.62%) patients, out of the total 189 patients (Fig. 4). This group had seven revision ACL reconstructions. Out of the 24 female patients, 20 (83.33%) required harvesting of both ST and G. In the weave-graft preparation group, the male and female patient number was 136 and 15, respectively (Fig. 4). Their mean age was 32.37 years (range, 19–47 years) (Table 1). ST + G harvest was required in only 38 patients (25.17%) and ST alone was sufficient in 113 patients (74.83%) out of the total 151 patients (Fig. 4). This group had four revision ACL reconstructions. Out of the 15 female patients, eight patients (53.33%) required harvesting of both ST + G (46.66%). On comparison of the proportion of the patients requiring only the ST graft, the *p* value came out to be highly significant (*p* < 0.0001), showing that the ST tendon alone is enough when using the weave technique to achieve the required graft diameter. Hamstring strength, measured by using prone knee bending, was initially better in patients in whom the G was preserved but both groups were able to lift a similar weight (8–10 kg) at the end of the rehabilitation program at 6 months after surgery. On radiographic laxiometry (anterior), performed immediately postoperatively and at 6 months after complete rehabilitation, the difference was insignificant between the two groups. It was between 2 and 4 mm for both the groups with average being 2.5 mm in both groups. Subjectively, at 2-year follow-up, there was no difference between the two groups in terms of functional outcome that was measured using IKDC 2000 (parallel-graft group mean: 89.61 ± 12.65 vs. weave-graft group: 87.55 ± 10.63; *p* = 0.1105 and SE = 1.288). The rerupture rate of the reconstructed ACL was higher in the parallel-strand graft (five cases, 2.64%) compared to the weave graft (two cases, 1.32%) at the end of the 2-year follow-up, but the difference was not statistically insignificant (*p* value = 0.3945) (Table 2).

**Table 1:** Demographics of the study

Serial number	Demographic value	Parallel-technique group	Weave-technique group
1.	Gender (total)	189	151
	Male	165	136
	Female	24	15
2.	Age		
	Mean	32.2 years	32.37
	Range	20–45 years	19–47

**Table 2:** Comparison of clinical results between the two groups

Serial number		Parallel-technique group	Weave-technique group	*p* value
1.	IKDC 2000 score (mean at 2-year follow-up)	89.61	87.55	0.1105
2.	Rerupture of reconstructed ACL	5 (2.64%)	2 (1.32%)	0.3945
3.	Radiographic laxiometry (at 6 months)	2–4 mm	2–4 mm	

*ACL* anterior cruciate ligament, *IKCD* International Knee Documentation Committee

## Discussion

Our results indicate that with the weave technique for graft preparation, similar diameter and functional outcome can be achieved compared to the parallel-strand technique and the frequency of G tendon harvest is reduced.

Interstrand healing was studied by Yan Xu in a rabbit model. This study showed that the four- strand hamstring tendon needs to pass through the necrosis, revascularization, and ligamentization stages, but the different strands are not involved in a synchronous process. The interstrand gap may be completely fused, partially fused, fused but connected with connective tissue, or still separated. By braiding the strand, the fusing percentage of the graft could be elevated and biomechanical properties could be improved [12]. This suggests that in the parallel-strand technique, the strands work as individual strands whereas in the woven strand configuration, all strands work together [12]. But weaving differs from braiding as it does not involve twisting and two strands remain parallel and only one strand is woven over the others, which helps all three strands to bind to each other strongly.

Spragg et al. have shown that the appropriate graft diameter is within the range of 7–9 mm and there is a 0.82-times lower likelihood of a revision with every 0.5-mm incremental increase in the graft diameter [13, 14]. Therefore, we made a protocol at our institute to achieve a 7–10-mm graft diameter. This diameter can be achieved without using the G in most cases if the weave technique is used. In our study, we also found that the rerupture rate was lower in the weave-technique group compared to the parallel-strand group; however, a longer follow-up is required because of the statistical insignificance.

As per the study by Waly, ACL reconstruction using a triple ST tendon is a viable alternative, preserving the G tendon and decreasing hamstring morbidity [15]. In a study by Tashiro et al., patients with quadrupled hamstring graft had some postoperative weakness with deep knee flexion due to loss of the G [16]. Therefore, if the G is preserved, it helps in early rehabilitation. Stengel et al. used a triple-strand hamstring graft and showed that laxity after surgery was due to the fixation method (i.e., due to screws and pins, not due to graft) [17]. Goradia et al. used a triple-strand hamstring graft in their study and showed that 90% of patients could be expected to have a normal or nearly normal knee at short- to intermediate-term follow-up [18]. Many authors suggest that good results can be obtained with a triple-strand hamstring graft by not using the G tendon to prevent flexion weakness of the knee. We used the triple-strand ST tendon with a different graft preparation technique (weave technique), which resulted in preservation of the G in majority of cases.

In order to assure the optimal 8-cm length and 7-mm thickness of the triple-strand hamstring graft construct for ACL reconstruction, it is essential to obtain a minimum tendon length of 24 cm. We used the new technique for hamstring graft preparation called the weave technique in this study. In this technique, two parallel strands of the hamstring graft, mostly the ST tendon, are woven together by the last third of the strand of the tendon winding around two tendons at an oblique angle with tension applied at the end before securing all three strands together. This is different from braiding where all three strands are twisted over each other; in the weave technique, the two strands of the ST remain parallel and the third strand are woven together by the remaining one third of the tendon. With proper pretensioning of the graft, there are no loose spaces between the strands that can cause thinning and loosening of the graft later.

There is no clear consensus among previous biomechanical studies done using animal tendons to determine the effect of braiding or twisting on graft strength and stiffness. Tis et al., in a published study, demonstrated that braiding caused a significant decrease in the strength and stiffness of human hamstring tendon tissue [19]. By contrast, till now, no study has been done on the weave technique. So, we conducted an in vitro analysis of a goat tendon preparation to compare stress and strain between the weave and parallel-strand techniques. In the study, the parallel-bundle graft and woven-bundle graft were subject to cyclic loading and graded according to the increase in the force applied to them. The length and diameter of the strands used for both the parallel and woven grafts were similar. There was significant (6–7 mm) plastic deformation of the parallel-strand graft as compared to the woven graft at a 50-N force when applied in a graded manner. The study showed the greater strength of the graft in a woven pattern compared to the parallel-strand graft. The limitations of this study were the limited availability of the associated literature and the in vitro study design using goat tendons not human tendon tissue to measure quantitative difference in the stress and strain pattern of the woven strand.

It is very important to restore muscle strength after ACL reconstruction because the motion and stability of the knee are controlled not only by static stabilizers, such as ligaments, but also by dynamic stabilizers such as muscles. Coombs and Cochrane reported a deficit in knee-flexor strength that lasted for at least 12 months after ACL reconstruction with a combined ST and (GR) tendon graft, even after a full rehabilitation protocol was followed [20]. Ardern and Webster showed that hamstring strength deficits persisted for a mean of 32.5 months after ACL reconstruction, despite completion of a rehabilitation program [21]. However, they did not find significant differences between the STGR and ST groups in any of the measures used in their study. Inagaki et al. found no differences in knee stability and clinical outcome between the ST and (STGR) groups 2 years after ACL reconstruction [22]. We found in our study that during rehabilitation, flexor strength of patients in whom the G was preserved was greater initially than that of the patients in whom the G was harvested; however, at 6 months of follow-up, both groups had similar flexor strength.

Yosmaoglu and Baltaci compared the results of a multi-joint, lower-limb, tracking-trajectory test, the peak torque of knee extensors and flexors at 60/s and 1800/s, and anterior tibial translation in patients who received ST and STGR grafts at 12 months after ACL reconstruction. They found that the side-to-side differences in flexor peak torque at 600/s were significantly higher in the STGR group than in the ST group. The side-to-side differences in extensor peak torque did not significantly differ, suggesting that preservation of the GR might improve postoperative athletic function [23].

The ST and G muscles also contribute to internal shin rotation; thus, it has been suggested that harvest of these tendons can result in internal shin-rotation weakness. Although the G is not truly a hamstring muscle it is considered to be a muscle of the medial compartment of the thigh which flexes and medially rotates the shin at the knee. Interestingly, Ahlen and Liden, evaluating a group of patients with a mean time since ACL reconstruction (STGR) of 8.5 years, demonstrated significant side-to-side differences in flexion peak torque but no significant peak torque deficit in internal shin rotation. Because of significant weakness in deep knee flexion, the authors suggested avoiding STGR autografts for athletes who depend on strength in deep flexion [24]. The possibility of increased risk of re-injury due to internal shin-rotational weakness and clinical significance of internal shin rotation in sports requiring shin rotation have also been discussed by Armour and Forwell [25].

Short follow-up is one of the limitations of this study; another one is that the time period of the techniques was quite different. Future studies involving a longer follow-up and objective measurement of the maximum strength of human autografts prepared in either way are required.

## Conclusion

The weave technique for hamstring graft preparation in ACL reconstruction is a good alternative to the conventional parallel-strand technique using aperture fixation. The weave technique helps to reduce the need for G harvest without compromising the diameter of the graft, final functional outcome, and stability.

## Data Availability

The data that support the findings of this study are available from (hospital where operations were performed) but restrictions apply to the availability of these data, which were used under license for the current study, and so are not publicly available. Data are, however, available from the authors upon reasonable request and with permission of (hospital where operations were performed).
